# New Strategies for the Extraction of Antioxidants from Fruits and Their By-Products: A Systematic Review

**DOI:** 10.3390/plants14050755

**Published:** 2025-03-01

**Authors:** Kaio Vinicius Lira da Silva Bastos, Adriana Bezerra de Souza, Alessandra Cristina Tomé, Felipe de Moura Souza

**Affiliations:** 1 Department of Pharmacy, Universidade de Guarulhos (UNG), Guarulhos 07023-070, Brazil; kaiolbastos@gmail.com; 2 Department of Infectious and Parasitic Diseases, Universidade de São Paulo (USP), São Paulo 05508-220, Brazil; adriana.bsouza@yahoo.com; 3 Instituto Federal de Educação, Ciência e Tecnologia Goiano (IFGO), Campus Morrinhos, Morrinhos 75650-000, Brazil; alessandra.tome@ifgoiano.edu.br

**Keywords:** fruits, extraction techniques, antioxidants, phytochemicals

## Abstract

This review highlights the recent advancements in extraction techniques for bioactive compounds from natural sources, focusing on methodologies that enhance both efficiency and sustainability. Techniques such as pressurized hot water extraction (PHWE), solid-state fermentation (SSF), ionic liquids (ILs), and electrohydrodynamic (EHD) methods have shown significant potential in improving extraction yields while preserving the bioactivity of target compounds. These innovative approaches offer significant advantages over traditional methods, including reduced energy consumption, minimal environmental impact, and the ability to extract thermosensitive compounds. PHWE and EHD are particularly effective for extracting antioxidants and thermosensitive compounds, whereas SSF provides an environmentally friendly alternative by valorizing agro-industrial waste. Ionic liquids, although promising for extracting complex phytochemicals, face challenges related to scalability and economic feasibility. The adoption of these advanced techniques represents a shift toward more sustainable and cost-effective extraction processes, promoting the discovery and utilization of high-value compounds. These methods also contribute to the development of eco-friendly, cost-effective strategies that align with green chemistry principles and regulatory standards. However, further research and technological advancements are required to address existing limitations and ensure the widespread application of these methods in industrial and pharmaceutical sectors.

## 1. Introduction

Fruits and their by-products hold significant potential in research and industry, particularly for the discovery and identification of novel chemical compounds [1,2]. A substantial proportion of fruit production is discarded; however, when utilized for the extraction of bioactive compounds, these materials can serve as valuable and sustainable resources [1,2].

Global fruit waste has reached alarming levels, with recent estimates indicating that approximately 45–55% of all fruits produced are lost or wasted along the supply chain [3,4]. According to the Food and Agriculture Organization (FAO), this amounts to roughly 3.7 billion metric tons annually, posing considerable environmental and economic challenges [5]. In developing countries, losses primarily occur during post-harvest handling and processing, whereas in developed nations, retail and consumer waste are the predominant issues [3,4].

Dietary and bioactive antioxidants, which are compounds present in fruits and their by-products, are found in various parts of the fruit, including the pulp, seeds, and even the peel [1,5,6]. Different parts of the fruit offer unique types and concentrations of antioxidants [1,5]. The extraction of antioxidants from the peel, pulp, and seeds is a common method used in food science and research to investigate their potential health benefits [1,5,6]. These compounds are frequently used in the development of supplements, functional foods, or cosmetic products [5,6].

Over the past decade, extraction technologies have undergone significant advancements, with a notable shift toward more sustainable approaches [5,6]. Traditional solvent-based extraction methods, which dominated the field until the early 2000s, have gradually been replaced by more sophisticated techniques that prioritize both efficiency and environmental sustainability [7,8]. This transition has been driven by an improved understanding of bioactive compound stability, increasing demand for natural products, and the implementation of stricter environmental regulations [9]. Key developments include the optimization of pressurized liquid extraction parameters, the integration of enzyme-assisted processes, and the introduction of novel green solvents [7,8].

Despite these advancements, a significant knowledge gap remains regarding the comparative effectiveness and scalability of these emerging extraction methods [9,10]. While numerous studies have investigated individual techniques, comprehensive analyses systematically comparing these methods across different fruit matrices and target compounds are still lacking [10,11]. Additionally, the economic feasibility and industrial applicability of these novel approaches require thorough evaluation [12].

The selection of an extraction method depends on factors such as the compound profile within the extract, enhancement of bioactivity, and optimization of extraction efficiency [13]. Given that different extraction techniques yield varying concentrations and phytochemical profiles, the extraction process plays a pivotal role in determining both the chemical composition and bioactivity of natural extracts [2]. Moreover, the extraction process influences the concentration of alkaloids, flavonoids, phenols, and tannins in the extracts, ultimately affecting their biological and antioxidant activities [2].

The concentration and variability of these bioactive compounds are intrinsically linked to the extraction method and the intended applications of the extracted materials [2]. Notably, advancements in phytochemical extraction technologies have not only improved yield optimization but also preserved the chemical and biological integrity of the compounds. New extraction strategies have shown promise due to their ability to reduce solvent and energy consumption, making the process more sustainable and environmentally friendly [1,2,3,4]. These advancements represent a crucial milestone in the transition toward greener extraction practices [1,2]. Consequently, recent studies have increasingly focused on integrating traditional techniques with emerging technologies to maximize extraction efficiency [1].

This review aims to explore the phytochemical and antioxidant extraction methods that have revolutionized the field over the past five years, with a focus on improving yields and proposing more efficient extraction strategies for bioprospecting-related research, while examining new techniques and methodologies. The review protocol has been publicly available on the Open Science Framework and is registered under DOI: 10.17605/OSF.IO/QZ6VM.

## 2. Materials and Methods

**Figure 1:**
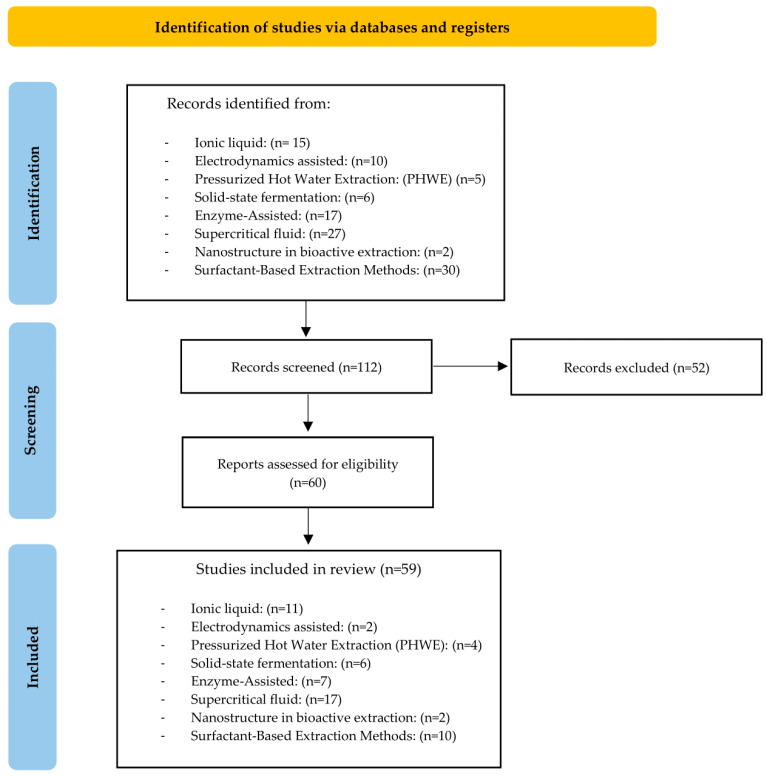
Preferred Reporting Items in Systematic Reviews and Meta-Analyses (PRISMA) flow diagram of inclusion and exclusion of peer-reviewed studies on this systematic review. The “*n*” denotes the number of articles identified and included through the selection process.

This systematic review followed the guidelines outlined in the Preferred Reporting Items for Systematic Reviews and Meta-Analyses (PRISMA). Following an impartial review of all the titles and abstracts obtained, two authors chose papers that satisfied the requirements for inclusion. These comprised human peer-reviewed research articles of various kinds (Figure 1 illustrates the research findings).

The electronic database search was conducted in Scopus (as of 29 November 2024). The search terms included all combinations of the following keywords:

“Antioxidants” AND “Extraction” AND “Extraction Type” AND “Phytochemicals”.

For each section that addressed an extraction method, the descriptor “Extraction type” was changed, and a specific descriptor was used for each section.

- **Inclusion Criteria**

The inclusion criteria were as follows: (a) papers from the last 5 years were included; (b) the researchers should address at least one of the phenolic quantification tests or antioxidant capacity tests (TPC, DPPH, ABTS, or FRAP); and (c) only studies published in the English language were taken into consideration.

All the included articles were thoroughly reviewed and analyzed.

- **Exclusion Criteria**

We excluded experimental papers if, after reading the title, abstract, or full text, they did not address the investigated methodology.

## 3. Results and Discussion

### 3.1. Primary Methodologies

#### 3.1.1. Pressurized Hot Water Extraction (PHWE)

Pressurized hot water extraction (PHWE) is an innovative technique for extracting phytochemicals, such as phenolic compounds, flavonoids, and other secondary plant metabolites [14,15]. This methodology leverages the properties of water when heated to high temperatures (typically between 100 °C and 250 °C) under pressure, preventing boiling and enhancing its solvency power [15,16,17].

The technique uses a green solvent—pressurized hot water—applied to plant material to extract compounds, often outperforming conventional extraction methods [16]. The efficiency of PHWE depends on several variables, such as flow rate, applied temperature, solvent system, and particle size [14,15,16,17,18]. However, the applied temperature is a critical factor influencing the yield and extraction of each compound [17].

Tan and colleagues [14] evaluated the relationship of temperature to assess the efficiency of PHWE in producing mangosteen pericarp extract using three marker compounds—α-Mangostin, γ-Mangostin, and Gartanin. The analysis revealed that extraction temperatures significantly influence the chemical profiles, resulting in distinct chemical profiles at different temperatures. It was observed that the concentration of α-Mangostin was lowest at 60 °C, increasing significantly at temperatures between 80 °C and 120 °C [14]. In turn, the concentration of γ-Mangostin was similar between 60 °C and 100 °C but peaked at 120 °C [14]. As for Gartanin, it was only detected at temperatures of 100 °C and 120 °C, with maximum yields under these conditions, emphasizing the strong relationship between temperature and the target compound to be extracted [14].

Another relevant factor related to temperature is antioxidant capacity. In accordance with Ong and colleagues [17], Tan and colleagues [14] found higher antioxidant activity in extracts obtained at 100 °C and 120 °C. The IC_50_ of the extract obtained at 120 °C was significantly lower than at lower temperatures, indicating greater antioxidant potency. This factor may be particularly relevant for producing more efficient antioxidant extracts [16,17].

However, the positive relationship between temperature and antioxidant capacity is not observed in the study by Ong and colleagues [17]. The antioxidant activities of avocado seed extracts were evaluated using DPPH and ABTS assays to investigate the effect of temperature on antioxidant capacity. The samples were extracted at different temperatures (60 °C, 80 °C, 100 °C, and 120 °C). The study also compared the extracts obtained at 60 °C, 80 °C, 100 °C, and 120 °C with those extracted by sonication with methanol [17]. Although the number of compounds extracted increased from 60 °C to 100 °C, no significant differences between the temperatures were observed. The extraction efficiency by PHWE at 80 °C was comparable to or greater than sonication with methanol [17]. In the DPPH assay, the extracts at 60 °C and 80 °C showed significantly lower IC_50_ values (*p* < 0.05) compared to those extracted at 100 °C and 120 °C. In the ABTS assay, the extracts at 60 °C and 80 °C also exhibited higher CEAC values, indicating greater antioxidant capacity. This highlights that each fruit has its optimal temperature range for extraction [17]. The results confirm that the PHWE method, especially at lower temperatures (60 °C and 80 °C), is effective for extracting bioactive compounds with strong antioxidant activity from avocado seeds [17]. Previous studies have reported a significant ability to obtain extracts with enhanced antioxidant activity, demonstrating performance comparable to conventional solvent extraction techniques [18].

Ong and colleagues [15] aimed to use PHWE to obtain phytochemicals from okra (*Abelmoschus esculentus* L. Moench). The extraction was performed at 60 °C, 80 °C, 100 °C, and 120 °C to identify the optimal extraction temperature, considering the phytochemical profile, antioxidant activities, and antidiabetic properties [15]. The results indicated that 80 °C was the ideal temperature for PHWE extraction, with the seeds having the highest concentration of quercetin and other antioxidant compounds. Additionally, seed extracts exhibited the lowest IC50 values for DPPH and ABTS assays, indicating greater antioxidant potential [15].

Another significant finding reported was that the okra seed extract significantly inhibited the α-glucosidase enzyme, achieving efficacy comparable to the antidiabetic drug acarbose [15]. PHWE proved to be an efficient and sustainable method for extracting bioactive compounds from okra, particularly from the seeds, which exhibited promising antioxidant, antidiabetic, and anti-inflammatory properties [15]. These results highlight the potential of okra seeds as natural ingredients for functional foods and pharmaceutical applications [15].

PHWE has proven to be a sustainable and solvent-free method for producing antioxidant extracts from black and red quinoa [16]. The distinct chemical profiles observed correlated well with their antioxidant activities and cytoprotective effects [16]. The analysis revealed that seeds extracted at 80 °C have distinctive chemical profiles, while extractions at 100 °C and 120 °C showed greater similarity [16]. Their research further highlighted that the type of fruit processing (pulverization) and extraction conditions (temperature) directly influence the quality and bioactivity of quinoa extracts, providing insights to optimize their commercial and nutritional applications [16].

Under high temperature and pressure, the polarity of water decreases, facilitating the extraction of less polar compounds [14,15]. In this way, PHWE can extract a wide range of phytochemicals without using harmful solvents [14,18]. The technique generally allows for shorter extraction times and high recovery rates of compounds, saving time and reducing the degradation of heat-sensitive phytochemicals [17,18].

Although high temperatures increase the solubility of compounds, the polarity of water decreases under high temperature and pressure, facilitating the extraction of less polar compounds. However, this condition can also lead to the degradation of heat-sensitive phytochemicals, such as certain flavonoids and vitamins [14,18]. Therefore, maintaining strict control over temperature, pressure, and extraction time is crucial, as even small variations can significantly affect both the quantity and quality of the extracted compounds [4].

PHWE requires specialized equipment, such as pressurized extractors, to maintain water in a liquid state under high temperatures and pressures, making it more expensive than conventional extraction methods [15]. However, this requirement has not yet contributed to a reduction in overall costs [15].

The PHWE has been successfully applied to extract bioactive compounds from various plant sources, such as fruits, herbs, spices, and agro-industrial residues [14,15]. In studies on antioxidant extraction, PHWE has proven to be efficient in extracting phytochemicals from diverse sources such as leaves, fruit peels, and a variety of plant residues, providing extracts with high antioxidant potential and anti-inflammatory activity [16].

The development of PHWE for phytochemical extraction holds promise for advancements in phytochemical research and the natural ingredients industry. Further studies are required to optimize temperature, extraction time, and pressure to minimize the degradation of sensitive compounds. PHWE presents a promising and sustainable alternative for phytochemical extraction, offering significant advantages in terms of efficiency, environmental safety, and potential industrial applications.

#### 3.1.2. Solid-State Fermentation (SSF)

Solid-state fermentation (SSF) is a biotechnological process that involves cultivating microorganisms on a solid substrate with sufficient moisture to allow fermentation [19]. This process differs from submerged fermentation in that the substrate is neither submerged nor dissolved in water [20]. In SSF, microorganisms grow on the surface of solid particles or a fibrous matrix, utilizing the moisture present in the solid substrate [20,21].

SSF employs solid substrates, which may include organic materials such as wheat bran, rice husks, sugarcane bagasse, and various fruit or food residues [19,20]. This process has gained increasing industrial interest due to its efficiency, low cost, and reduced environmental impact, particularly to produce enzymes, antibiotics, bioactive compounds, and biofuels [19].

Filamentous fungi are the most used microorganisms in SSF, although bacteria and yeasts can also be employed, depending on the desired product and the metabolic characteristics of the species [19,20]. Among these, *Aspergillus niger* is widely utilized due to its efficiency in biomass conversion and bioactive compound production [19,20]. Fungi play a crucial role in SSF because they produce extracellular enzymes that degrade the substrate into smaller molecules, which they then absorb for growth and metabolism [19]. By breaking down the substrate and releasing phenolic compounds or phytochemicals bound to plant structures, fungi generate valuable bioactive products. This process not only facilitates the extraction of previously inaccessible substances but also reduces antinutritional factors in the substrate [19,21].

A notable example is the study by Suri and colleagues [20], which evaluated the debittering of Kinnow mandarin peels through solid-state fermentation (SSF). The SSF process employed *A. niger* koji to assess the efficiency of naringin hydrolysis, a bitter antinutritional compound. The study found that submerged fermentation resulted in the lowest naringin content (5.09 mg/g) but also yielded lower values for total phenolic content (TPC, 28.11 mg GAE/g) and total antioxidant activity (TAA, 45.99%) [20]. In contrast, SSF was more effective in extracting phenolic and antioxidant compounds, with TPC values of 25.33 mg GAE/g, TAA of 44.15%, and a significant reduction in naringin content to 7.08 mg/g [20]. When compared to chemical debittering, which also reduced naringin content (6.57 ± 0.33 mg/g), SSF proved superior in preserving the natural composition of total phenolics and antioxidant capacity. Chemical debittering resulted in lower TPC (21.16 mg GAE/g), total flavonoid content (TFC, 10.59 mg QE/g), and TAA (34.36%) [20]. These findings highlight the potential of Kinnow mandarin peels, post-debittering, as functional ingredients in food and confectionery products, enhancing their nutritional value with bioactive antioxidants, dietary fibers, micronutrients, and pectin, without residual bitterness [20].

Chen and colleagues [19] demonstrated that SSF using *A. niger* enhances the extraction of polyphenols bound to plant structures. The SSF-derived extract exhibited greater antioxidant activity than the extract obtained via alkaline hydrolysis of *Rosa roxburghii* fruit pomace insoluble dietary fiber [19]. Throughout the fermentation process, a continuous release of bound phenolics was observed, with the highest TPC concentration recorded on the third day [19]. However, after this period, TPC levels declined, likely due to nutrient depletion, fungal stress responses, and the degradation or polymerization of phenolic compounds [19]. This finding underscores the third day as the optimal extraction period for this substrate [19]. The study confirms that SSF with *A. niger* provides an efficient and environmentally friendly approach to releasing bound polyphenols from *R. roxburghii* fruit pomace [19].

Mane, Anand, and Vedamurthy [21] investigated SSF for bioprospecting bioactive compounds from corn bran—*Zea mays*—using endophytic fungus *Thielaviopsis basicola*, isolated from Ximenia americana in the Western Ghats of Karnataka, India. Fermentation was conducted under dark conditions at 25 ± 2 °C for 15 days [21]. The study identified and purified bioactive compounds, offering valuable insights for the extraction of novel chemical compounds and pharmaceutical development [21]. A total of 32 fungal endophytes were isolated and evaluated for antioxidant activity, demonstrating high antioxidant capacity, with 86.2% inhibition in the DPPH assay [21].

Purewal and colleagues [22] examined the effect of SSF on pearl millet grains, fermenting them for 336 h using *Aspergillus oryzae* and *Rhizopus azygosporus*. The fermented millet (koji) was extracted under optimized conditions, and its bioactive compounds were analyzed via high-performance liquid chromatography (HPLC) [22]. In non-fermented grains, the predominant compounds were ascorbic acid (1.55 mg/g), p-coumaric acid (1.04 mg/g), and cinnamic acid (0.71 mg/g) [22]. Fermentation with *A. oryzae* significantly increased these compounds, yielding ascorbic acid (10.23 mg/g), gallic acid (8.95 mg/g), resorcinol (2.90 mg/g), catechol (7.60 mg/g), vanillin (4.58 mg/g), p-coumaric acid (3.96 mg/g), quercetin (2.74 mg/g), benzoic acid (5.10 mg/g), and cinnamic acid (5.14 mg/g) [22]. Similarly, millet fermented with *R. azygosporus* exhibited enhanced levels of ascorbic acid (8.80 mg/g), gallic acid (4.70 mg/g), and p-coumaric acid (1.27 mg/g) [22]. Both fermented extracts demonstrated excellent DPPH inhibition (>96.12%), confirming SSF as an effective strategy for antioxidants extraction [22].

Yepes-Betancur and colleagues [23] evaluated the SSF of avocado seeds with *A. niger* GH1 to release bioactive compounds with antioxidant capacity. The optimal conditions identified included a particle size of 2.5 mm, 60% moisture, and 120 h of fermentation, resulting in the highest TPC release (14.56 mg GAE/g) [23]. Antioxidant capacity assays (ABTS and DPPH) showed significant increases (33% and 77% with water; 28% and 42% with culture medium), with the highest antioxidant potential observed in the DPPH assay after 168 h [23]. These findings highlight the role of SSF in enhancing the antioxidant potential of avocado by-products, contributing to the sustainability of the avocado industry [23].

SSF presents technical challenges, particularly in controlling temperature, humidity, and pH, as solid substrates retain heat and moisture unevenly [20,21,22,23]. Oxygen and nutrient diffusion may also be limited, affecting microorganism growth and metabolite production [22,23,24]. Additionally, extracting and purifying final products remains complex and costly [19,20].

Advancements in bioprocess technology have enhanced the efficiency and competitiveness of SSF across various industries [22,23,24]. Current research focuses on optimizing process conditions, refining substrate selection, and developing genetically modified microorganisms to improve enzyme and metabolite yields [19].

Agro-industrial residue as substrates is being investigated considering the growing interest in sustainability and the circular economy [25]. SSF represents a powerful tool in modern biotechnology, offering industrial, economic, and environmental benefits. Although technical challenges remain, its potential for transforming waste into high-value products makes it a strategic technology for multiple industries.

#### 3.1.3. Ionic Liquid Extraction

Ionic liquids (ILs) emerge as promising and sustainable alternatives for the extraction of chemical compounds from fruits and vegetables [26,27]. These solvents are salts with low melting points and remain in the liquid phase under normal conditions [28,29]. Their ionic nature enables unique properties such as high thermal stability and low volatility, allowing for the adjustment of their characteristics to enhance selectivity and extraction efficiency [30]. The selectivity of ILs is based on specific interactions between bioactive compounds and the chemical components of the ILs [27,28,29,30]. The nature of the cations and anions in an IL can directly influence solubility, polarity, and complex formation with different bioactive compounds, such as flavonoids or carotenoids [27,28,29,30]. These chemical interactions are essential for optimizing extraction efficiency and process selectivity, as the choice of IL must be based on the physicochemical properties of the target bioactive compound [27,29]. To promote the use of these solvents as greener and more sustainable techniques, their lower toxicity and reduced environmental impact compared to conventional organic solvents may facilitate the replacement of traditional extraction strategies [27].

This recent technique has demonstrated excellent efficiency in the extraction of β-carotene, as described by Ferreira and colleagues [26]. Their study evaluated the extraction of β-carotene from buriti fruit (*Mauritia flexuosa*) using ionic liquids as an alternative to conventional organic solvents like acetone [26]. The extraction with 1-butyl-3-methylimidazolium tetrafluoroborate [C_4_mim][BF_4_] not only improved β-carotene extraction efficiency compared to acetone (19.21 and 13.13 mg/100 g, respectively) but also provided greater pigment stability during light, thermal, and color stability tests [26]. Furthermore, the use of ILs such as [C_4_mim][BF_4_] demonstrated superiority in β-carotene extraction from buriti, offering better stability under light and heat while reducing environmental impact [26]. These findings highlight its potential as a green and sustainable solvent for the food industry, improving the stability and safety of natural colorants [26]. Other studies have also achieved efficient vegetable carotenoid extractions, including investigations with orange peel [29] and *Bactris gasipaes* fruit waste [28,31].

Dimitrijevic and colleagues [27] developed an integrated approach using aqueous biphasic systems (ABS) with choline-based ionic liquids and PPG400 to extract, separate, and concentrate polyphenols (resveratrol, quercetin, and gallic acid) from grape extracts. Among the seven ILs tested, [Ch][DHP] exhibited the highest extraction efficiencies (>95% for resveratrol and quercetin and 90% for gallic acid) and excellent selectivity for separating gallic acid from other polyphenols [27]. The protocol by Dimitrijevic and colleagues [27] achieved 100% recovery of resveratrol, reinforcing the sustainability of this type of extraction and enhancing the economic feasibility of chemical-industrial processes [27].

Du and colleagues [30] compared the solid-phase dispersion extraction method with a β-CD matrix assisted by IL [30]. The developed method was successfully applied to extract and determine active compounds in Mori Fructus samples, achieving analyte recoveries ranging from 93.5% to 98.3% [30].

The recovery or extraction of chemical compounds from food waste is becoming increasingly efficient, as highlighted by the research of Faria and colleagues [32]. This study investigated aqueous solutions of ionic liquids (ILs) with hydrotropic or surfactant properties to enhance solubility and effectively extract syringic acid from pear peels [32]. The results showed that ILs with cationic hydrotropic behavior significantly increased the solubility of syringic acid by up to 84 times compared to an aqueous solution [32]. The reuse of the solvent and biomass increased the yields to 2.04% and 2.22%, resulting in a recovery of syringic acid of 77% of the extracted compound [32].

The study by Ali and colleagues [33] demonstrated the efficiency of this extraction method through in vitro clinical studies. The fruit extract of *Mallotus philippensis* showed significant antimicrobial activity against *Pseudomonas aeruginosa* and *Escherichia coli* and exhibited high antioxidant capacity, achieving 97% inhibition in the DPPH assay [33]. These results highlight the potential of the ionic liquid-loaded microcapsules for efficient phytochemical extraction, particularly of alkaloids, flavonoids, and tannins, which are known for their pharmacological properties [33].

A sustainable solution was proposed by Das and colleagues [25], who utilized fruit and vegetable peel waste combined with graphene oxide composites (GOMIP-A) and the ionic liquid 1-allyl-3-octylimidazolium chloride (A) to selectively extract 4-hydroxybenzoic acid (4HA) [25]. This approach demonstrated high recovery efficiency and strong antioxidant potential of the extracted compounds [25].

The method developed by Ran and colleagues [34] extracted proanthocyanidins from grape seeds using an ethanol/(NH_4_)2SO_4_-based aqueous two-phase system (ATPS) with ionic liquids as additives. The study highlighted that this method outperformed traditional organic solvents (such as methanol, ethanol, and isopropanol) in extraction yield, increasing efficiency from 47.89–52.42% to 97.23–97.79% with the addition of 5 wt% [C_6_mim]BF_4_ to the ATPS [34].

The microwave-assisted extraction method using ionic liquids proposed by Handayani, Wulan, and Abdul [35] demonstrated greater efficiency in extracting phenolic compounds (quercetin, mahkoside, benzophenone, and mangiferin) and higher selectivity for polar compounds compared to reflux and Soxhlet extraction methods for the fruit *Phaleria macrocarpa* [35].

Although the use of ionic liquids on a small scale has shown excellent results in terms of extraction efficiency, transitioning to an industrial scale still presents significant challenges. The application of ILs in industrial processes requires adaptation of equipment, especially due to their high viscosity and the need for corrosion-resistant materials. Additionally, economic issues related to the production cost of ILs and the need for additional purification processes to obtain high-purity compounds are barriers to commercial viability. Innovations such as combining ILs with auxiliary techniques, such as microwave-assisted extraction, have shown potential to overcome these challenges and improve overall process efficiency.

Ionic liquids as solvents in phytochemical extraction have garnered increasing attention due to their ability to extract compounds such as flavonoids, alkaloids, terpenes, and other secondary metabolites with high efficiency while preserving their bioactive properties. Despite these challenges, extraction with ionic liquids offers an innovative and more sustainable solution to maximize the yield and quality of bioactive antioxidant compounds extracted from natural sources.

#### 3.1.4. Electrohydrodynamic-Assisted Extraction (EHD)

Electrohydrodynamic-assisted extraction (EHD) is an innovative technique that utilizes high-voltage electric fields to enhance the extraction of bioactive compounds from natural materials such as plants, microalgae, and agricultural residues [36,37,38]. This method integrates electrohydrodynamic principles, which govern the interaction between fluids and electrical forces, to improve the release of secondary metabolites and other intracellular compounds [39].

During the extraction process, the applied electric field generates forces that enhance solvent movement and induce structural changes in cell membranes, primarily through electroporation [37,38]. Electroporation temporarily forms pores in the membranes, facilitating the diffusion of bioactive compounds into the solvent [36,37,38]. As a result, mass transfer is significantly improved, leading to reduced processing times and, in many cases, minimizing the need for high temperatures or large solvent volumes [36,37,38].

EHD-assisted extraction offers distinct advantages in terms of efficiency, sustainability, and broad applicability across various matrices [36,37]. By operating under milder conditions, it is particularly well-suited for heat-sensitive compounds such as antioxidants, pigments, and essential oils [39]. Consequently, this technique presents a promising alternative to conventional methods, providing faster, more environmentally friendly, and cost-effective approaches for the extraction of natural products [36,37,38].

A significant study by Shahram and Dinani [36] investigated the feasibility of using orange pomace for the extraction of phenolic compounds. The effects of EHD treatment duration—evaluated at four levels (2, 10, 20, and 30 min)—and EHD voltage—tested at four levels (0, 14, 18, and 22 kV)—were assessed in relation to total phenolic content (TPC), antioxidant activity (AA), and extraction efficiency (EE) [36]. The results indicated that the optimal extraction conditions were achieved with a treatment time of 10 min and a voltage of 18 kV, yielding TPC, AA, and EE values of 617.76 ± 6.15 mg/L, 91.97 ± 0.43%, and 64.00 ± 0.00%, respectively [36].

Similarly, Maher, Dinani, and Shahram [37] explored the extraction of phenolic compounds from lemon processing waste powder using EHD. The study examined two independent factors—EHD duration (10, 20, and 30 min) and EHD voltage (0, 15, and 19 kV)—as well as their combined effect on dependent variables such as TPC, AA, and extraction yield [37]. The results demonstrated that increasing the EHD duration from 10 to 30 min and voltage from 0 to 19 kV significantly enhanced both TPC and extraction yield [37]. Fourier-transform infrared (FTIR) spectroscopy confirmed that the EHD process did not degrade the functional groups of the extracted compounds, aligning with findings from Shahram and Dinani [36].

While EHD-assisted extraction has demonstrated significant potential in improving mass transfer and enhancing the recovery of bioactive compounds from plant matrices, scaling this technology for industrial applications presents notable challenges [36]. A primary limitation is its energy efficiency [36,37]. Although EHD improves extraction yields at the laboratory scale by facilitating solvent penetration and disrupting plant cell structures, its application to large-scale systems is hindered by high energy consumption [36,37]. Furthermore, the effectiveness of EHD varies depending on the electrical conductivity of the solvent and plant material, requiring careful optimization for each specific application [37].

These findings suggest that EHD is particularly effective for extracting thermosensitive compounds [36,37]. Beyond its ability to extract natural bioactive compounds such as phenolics, EHD represents a promising approach for the sustainable valorization of agro-industrial by-products, aligning with green chemistry principles and the growing demand for eco-friendly extraction technologies [36,37,38].

Electrohydrodynamic-assisted extraction (EHD) has emerged as a promising and sustainable technique for recovering bioactive compounds from various natural sources. By utilizing high-voltage electric fields to enhance mass transfer through electroporation, this method offers significant advantages over conventional extraction techniques, particularly for thermosensitive compounds. Studies have demonstrated its efficiency in improving extraction yields while operating under milder conditions, thereby reducing solvent consumption and processing time [36,37,38].

Despite its potential, large-scale implementation of EHD remains challenging due to high energy requirements and the need for precise optimization based on the electrical properties of different matrices. Addressing these limitations through advancements in energy-efficient designs and process integration will be critical for industrial adoption. Nonetheless, the alignment of EHD with green chemistry principles underscores its potential to contribute to more sustainable extraction processes, particularly in the valorization of agro-industrial by-products.

#### 3.1.5. Enzyme-Assisted Extraction (EAE)

Enzyme-assisted extraction (EAE) is a technique characterized by mild extraction conditions, with almost no negative environmental consequences [40]. It is considered a green extraction technique with commercial potential, making it suitable for sensitive environments with minimal ecological impact [41].

The EAE technique offers numerous advantages, including higher extract purity levels, reduced mechanical damage to equipment, especially in the extraction of pigments with undesirable colors [42], and the fact that it does not require expensive equipment [40]. One of the primary benefits of EAE is its potential to enhance the extraction of bioactive compounds through enzymatic disruption of cell wall integrity, facilitating the release of target compounds [41]. The efficiency of EAE depends on various functional parameters, such as extraction duration, relatively mild reaction temperatures (generally around 40–50 °C), enzyme concentration, pH level, and substrate particle size [43].

The most used enzymes in EAE are cellulase, pectinase, hemicellulase, and β-glucosidase due to their specific and effective properties, as well as their ability to break down plant cell compositions and disrupt membrane integrity, thereby improving the release of bioactive compounds [44]. These enzymes can be derived from various sources, including bacteria, fungi, fruit and vegetable extracts, or animal organs [45].

According to Sasikumar and colleagues [46], combining EAE with other techniques, such as microwave-assisted extraction (MAE), can maximize extraction efficiency and quality by synergistically enhancing the process and enabling the production of phytochemical-rich extracts [46]. Granato and colleagues [47] studied the effects of commercial enzymes (pectinases, cellulases, β-1,3-glucanases, and pectin lyases) on the extraction of polyphenols from blackcurrant (*Ribes nigrum*) press cake. Their results indicated that enzymatic treatment over an extended processing time increased antioxidant activity, as demonstrated by DPPH and CUPRAC assay results [47]. The combined treatment with β-glucanase and pectin lyase yielded superior results compared to the control treatment (without enzyme application) [47].

Machado and colleagues [48] investigated the enhanced recovery of antioxidant compounds from grape pomace (BRS Violeta) through cellulase-assisted extraction. Their findings showed that the presence of cellulase significantly reduced the antioxidant activity of grape pomace extracts, as assessed by DPPH and FRAP assays, compared to other extraction techniques [48]. The authors suggested that products of the enzymatic reaction might interfere with the analytical methods used, leading to an underestimation of the antioxidant properties of the extracts. However, further studies are needed to explore this hypothesis and identify potential interferences [48,49].

Sasikumar and colleagues [46] conducted a recent study on the integration of two emerging techniques, EAE and MAE, to synergistically enhance extraction yield and obtain phytochemical-rich extracts. Their research involved the use of pectinase at different concentrations in the extraction of wild Khasi cherry juice (*Prunus nepalensis*). The results showed that polyphenol content significantly increased through enzyme-assisted and microwave-assisted extraction [46]. The DPPH assay results indicated higher free radical scavenging activity with a treatment combining 600 W microwave power, 0.05% enzyme concentration, 60 min of enzyme incubation, and 195 s of microwave treatment [46]. Several studies have demonstrated that enzyme-assisted extraction (EAE) improves the yield and quality of bioactive compounds extracted from fruits (peel, pulp, and seeds) compared to traditional extraction methods [47,48].

Despite its many advantages, EAE presents certain limitations that must be considered. The effectiveness of enzymatic extraction is highly dependent on factors such as enzyme specificity, reaction conditions, and substrate composition, which can vary significantly between different plant matrices [45,46,47]. Additionally, enzyme activity may be inhibited by the presence of certain compounds in plant materials, reducing extraction efficiency [46,47]. Another challenge is the potential degradation of bioactive compounds caused by prolonged enzyme incubation times, which could affect the quality of the extract [45]. Moreover, the high cost of certain enzymes and the need for precise optimization of reaction parameters can limit the widespread adoption of EAE in large-scale industrial applications [45,46,47].

Enzyme-assisted extraction (EAE) is a promising and sustainable technique for obtaining bioactive compounds, offering advantages such as mild reaction conditions, high extract purity, and reduced environmental impact. However, limitations related to enzyme specificity, cost, and process optimization must be addressed to enhance its efficiency and applicability. Future research should focus on improving enzyme stability, optimizing reaction parameters, and combining EAE with other green extraction techniques to maximize its potential for industrial applications.

#### 3.1.6. Nanostructures

Nanostructures are a revolutionary tool in the field of extraction, providing unique features that enhance the efficiency, selectivity, and sustainability of bioactive chemical isolation [7,50]. Among them, biotechnology-based methods are an effective approach due to their alignment with the principles of green chemistry and sustainable development [6,7]. This approach is also economical, as it consumes less energy and does not require expensive reagents or organic solvents. Their high surface area-to-volume ratio, variable surface chemistry, and capacity to interact at the molecular level with target molecules make nanostructures particularly valuable in optimizing extraction procedures [50].

For instance, functionalized nanoparticles can selectively bind to specific molecules, enhancing the recovery of target compounds while minimizing contaminants [6,7]. Nanostructures can also improve the solubility and transport of bioactive compounds in solvent-based extractions [50]. The effectiveness and sustainability of both established and emerging extraction methods can be significantly enhanced by incorporating nanostructures into them [50,51,52,53]. High surface area, adjustable porosity, and customizable functionalization are just a few of the distinctive characteristics of nanostructures, allowing for more efficient and targeted interactions with specific molecules [52,53]. These advancements reduce the need for harsh chemicals, lower energy consumption, and generate less waste [52]. Furthermore, due to their versatility, nanostructures can be applied in various settings, such as the recovery of bioactive substances for pharmaceuticals or the separation of valuable components from industrial waste [53]. While enabling new approaches in resource recovery and the advancement of the circular economy, this innovative technique enhances overall efficiency [52,53].

Nanostructure-assisted extraction has demonstrated promise in several specific applications [50,51,52]. In fruit processing, functionalized magnetic nanoparticles have shown exceptional efficiency in recovering polyphenols from apple pomace, achieving extraction yields up to 95% higher than conventional methods [45,50,51,52]. Carbon-based nanostructures, particularly graphene oxide derivatives, have proven effective in the selective extraction of flavonoids from citrus peels, with enhanced specificity and reduced processing time [54]. Recent developments in metal-organic frameworks (MOFs) as extraction media have enabled the highly selective isolation of specific bioactive compounds [55]. For instance, zirconium-based MOFs have demonstrated superior performance in extracting anthocyanins from berry fruits, with extraction efficiencies exceeding 90% while maintaining compound stability [56]. These advances are particularly relevant for the pharmaceutical and nutraceutical industries, where high-purity extracts are essential [53]. Additionally, hybrid nanostructures combining different materials have emerged as powerful tools for multi-target extraction [50,51,52,53]. Silica-coated magnetic nanoparticles functionalized with specific ligands have shown remarkable versatility in simultaneously extracting different classes of compounds from complex fruit matrices, offering new possibilities for comprehensive bioactive compound recovery [55,56,57,58].

Despite the numerous advantages of nanostructure-assisted extraction, several limitations must be considered. One of the primary concerns is the potential toxicity and environmental impact of nanomaterials [55,58]. Certain nanoparticles, particularly those composed of heavy metals, may pose risks to human health and ecosystems if not properly handled or disposed of [54,55,56]. Additionally, the functionalization process required to enhance selectivity can be complex and costly, which may limit its widespread adoption [58]. Another challenge is the potential interference of nanostructures with the extracted bioactive compounds, which could alter their stability or bioavailability [58].

Nanostructure-assisted extraction is a promising approach that enhances the efficiency, selectivity, and sustainability of bioactive compound isolation. Its unique properties enable higher extraction yields and purity, benefiting industries such as pharmaceuticals, nutraceuticals, and food science. However, challenges related to toxicity, environmental impact, and scalability must be addressed to facilitate widespread adoption. Future research should focus on developing eco-friendly nanomaterials and cost-effective large-scale applications to maximize the potential of this innovative technology in sustainable extraction processes.

#### 3.1.7. Supercritical Fluid Extraction (SFE)

Supercritical fluid extraction (SFE) is an advanced extraction technology that employs various solvents as fluids under temperatures between 40 and 60 °C and pressures ranging from 200 to 400 bar, below their critical point [40]. This enhances the extraction of bioactive compounds from various materials due to their solvating properties [40,59]. Carbon dioxide (CO_2_) is the most used solvent due to its advantageous characteristics, including low toxicity, availability, and a comparatively low critical point [60].

Several studies suggest that using CO_2_ improves the efficiency of extracting bioactive compounds from fruits, preserving or even enhancing the nutritional value, safety, freshness, flavor, and health benefits of food products [61,62,63]. This makes it a standard extraction technology, particularly effective for valorizing fruit and vegetable residues due to the safety and purity of the natural extracts [61,62,63].

The advantages of extracting bioactive compounds from fruits using SFE surpass those of traditional extraction methods due to its high-quality output, cost-effectiveness, and environmental friendliness [62,63]. This has increased societal interest in the application of green and promising technologies [64]. Notable advantages include higher selectivity, greater purity of the extracts, elimination of solvent residues, and near-complete reuse of the CO_2_ solvent through process recirculation, which is considered an economic benefit [65].

However, supercritical CO_2_ has low polarity, which limits its ability to solubilize hydrophilic compounds. For more polar compounds, co-solvents such as methanol, ethanol, and water can be used as polarity modifiers to enhance the solubilization capacity for different classes of compounds [66]. Numerous studies have demonstrated the efficiency of co-solvents in recovering bioactive compounds [67,68,69,70]. Adding 1–20% of polar organic solvents (co-solvents), such as methanol and ethanol, can improve the relative permittivity, extraction selectivity, and solvent strength of CO_2_ [70].

Mota and colleagues [71] evaluated the influence of process variables (temperature and pressure) on the antioxidant activity of *Calycolpus goetheanus* extracts obtained via SFE-CO_2_, comparing the results with essential oils from *C. goetheanus* obtained through hydrodistillation (HD) [71]. The study aimed to assess the impact of extraction methods on the bioactive profile and antioxidant properties of *C. goetheanus* extracts [71]. The antioxidant activity results using the DPPH assay demonstrated that the extract obtained by SFE-CO_2_ at 35 °C/150 bar had the lowest IC_50_ value (120.54 ± 6.81 µg/mL) and the highest inhibition value (66.82 ± 0.81%), with no significant difference (*p* < 0.05) compared to the extract obtained at 45 °C/150 bar (125.35 ± 6.63 µg/mL) [71]. In contrast, the extract obtained via HD exhibited the highest IC_50_ value (334.87 ± 7.51 µg/mL) and the lowest inhibition percentage (25.04 ± 0.81%) [71]. The authors further reported that both HD and SFE-CO_2_ fractions effectively scavenged the ABTS radical, with significant differences (*p* < 0.05) observed among all results, ranging from 195.56 ± 6.44 mg Trolox/mL (HD extract) to 405.10 ± 9.31 mg Trolox/mL (SFE-CO_2_ extract at 35 °C/150 bar) [71]. These data support the advantages of SFE, including short extraction time, low energy consumption, and reduced solvent usage, as reported by various authors [71,72,73,74,75].

Grabauskaitė and colleagues [73] studied the extraction of compounds from the seeds and pomace of red fruits using supercritical fluid extraction (SFE-CO_2_) and pressurized liquid extraction (PLE). Comparing the antioxidant activity of the raw materials, they observed that ABTS•+ and CUPRAC values were slightly higher after SFE-CO_2_ application [72]. They hypothesized that the high-pressure SFE-CO_2_ method released insoluble polyphenolic antioxidants from the plant cell wall matrix, leading to higher antioxidant indices in the solid residues after extraction [72,73].

Reina and colleagues [74] explored the valorization of cashew nut shells (*Anacardium occidentale*) by extracting bioactive compounds using supercritical fluid extraction (SFE), subcritical water extraction (SCWE), and Soxhlet extraction (SE). Their results showed a significant difference in antioxidant activity measured by the DPPH assay, with SFE yielding the highest value (4.55 ± 0.09 Trolox/mL) compared to traditional SE (0.78 ± 0.15 Trolox/mL) [74]. The authors attributed these differences to the selective nature of the extraction methods, with SFE being the most selective for recovering phenolics from cashew nut shells [74].

Mihalcea and colleagues [75] applied supercritical fluid extraction (SFE) with CO_2_ to recover bioactive compounds from whole red grape pomace, including skins, seeds, and residual pulp. The extracts were characterized in terms of polyphenol, fatty acid, tocopherol, and phytosterol content [75]. The results showed that extracts with the highest polyphenol and carotenoid content were obtained at 45 MPa and 45 °C, while extracts obtained at 30 °C and 15 MPa exhibited higher fatty acid, tocopherol, and phytosterol content [75]. The authors concluded that increasing temperature and pressure improved the extractability of polyphenols and carotenoids, whereas lower temperature and pressure favored the extraction of fatty acids, tocopherols, and phytosterols [75].

Despite its numerous advantages, SFE has limitations that must be considered. One major challenge is the high initial investment cost associated with supercritical fluid extraction equipment, which can be prohibitive for small and medium-sized enterprises [61,62]. Additionally, the process parameters, such as temperature and pressure, require precise control to optimize extraction efficiency and prevent the thermal degradation of bioactive compounds [60,61,62,63]. Another limitation is the low polarity of CO_2_, which restricts its ability to extract polar compounds, necessitating the use of co-solvents that may compromise the “green” nature of the method [61,62]. Moreover, selecting optimal conditions for different matrices requires extensive preliminary studies, making the technique time-consuming and resource-intensive [61,62].

Supercritical fluid extraction (SFE) represents a highly efficient and environmentally friendly technique for obtaining bioactive compounds from plant matrices, particularly when using CO_2_ as a solvent. Its advantages, including high selectivity, solvent recyclability, and minimal thermal degradation of compounds, make it a promising alternative to conventional extraction methods. However, challenges related to cost, scalability, and the need for co-solvents must be addressed to facilitate broader industrial adoption. Future research should focus on optimizing operational parameters, improving energy efficiency, and integrating SFE with other sustainable extraction techniques.

#### 3.1.8. Surfactant-Based Extraction Methods: CPE and SUPRAS

In recent years, innovative surfactant-based extraction techniques, such as polymer-polymer cloud point extraction (CPE) and supramolecular solvents (SUPRAS), have shown significant potential for extracting bioactive compounds and antioxidants from fruits [76,77]. These emerging methodologies stand out for their efficiency, sustainability, and lower environmental impact compared to traditional methods that rely on organic solvents [76].

CPE has proven effective in recovering flavonoids and phenols from various fruits, including peaches and mandarin peels. Studies, such as that by More et al. [76], demonstrate that optimizing parameters like temperature and salt concentration, along with the use of Triton X-114 surfactant, enhances bioactive compound yields and antioxidant activity. This technique is particularly beneficial for preserving heat-sensitive compounds while minimizing organic solvent use, achieving superior results compared to conventional methods like ethanol extraction [76]. Furthermore, Cvanić et al. [77] demonstrated the potential of CPE in extracting antifungal bioactive compounds from horned melon peels, which exhibited inhibitory effects on fungal growth. This finding further highlights the versatility and effectiveness of CPE in both bioactive compound recovery and antimicrobial applications. It reinforces the adaptability of CPE for various plant-based matrices, showcasing its potential for applications beyond direct bioactive compound recovery [77]. In addition, Athanasiadis and colleagues [78] explored the antioxidant properties of lemon peel extracts using CPE, achieving high recovery of polyphenolic compounds, including eriocitrin and hesperidin, known for their beneficial health properties. This reinforces the adaptability of CPE for various plant-based matrices, showcasing its potential for applications beyond direct bioactive compound recovery [76,77,78]. Similarly, studies by Patel and colleagues [79] have shown that this technique can also be applied to other fruits, such as mandarin, effectively yielding phenolic compounds and flavonoids with high antioxidant potential. These findings further illustrate the versatility of the method.

On the other hand, SUPRAS, consisting of self-assembled supramolecular solvents, has proven to be more versatile, allowing the extraction of bioactive compounds of varying polarities, from phenols to carotenoids [80,81,82,83]. The study by Demir and colleagues [80] demonstrated the effectiveness of SUPRAS in extracting morin from fruits and beverages, highlighting its remarkable selectivity, which can be tailored to specific target compounds and fruit matrices. Additionally, this technique is recognized for its speed and efficiency, utilizing environmentally friendly solvents such as 1-dodecanol [80]. When combined with acids and ethanol, these solvents enable high yields of bioactive compounds without requiring high temperatures or toxic reagents [80]. This approach has been particularly useful for extracting antioxidant compounds from fruits like tamarillo and lemon peels, as demonstrated in studies by Krivošija and colleagues [81], which reported high yields of flavonoids and superior antioxidant activity for these fruit by-products [81]. This type of waste valorization highlights the potential of extraction techniques to promote sustainable practices in the food and nutraceutical industries [82].

However, SUPRAS presents challenges, particularly related to the complexity of the process [81,82,83]. Proper surfactant and solvent selection require careful optimization, which can make the process more time-consuming and costly [82]. Additionally, SUPRAS is often considered more complex than CPE, requiring a higher level of expertise in controlling variables such as temperature, solvent type, and surfactant choice, which may limit its scalability [80]. While CPE is more straightforward and less costly, it offers lower flexibility and selectivity [77,78,80]. The technique depends on the correct selection of surfactant and critical temperature, which can limit its use in complex matrices or for compounds that do not form stable micelles [83].

Moreover, the integration of SUPRAS with other techniques, such as ultrasonication and microwave radiation, has been explored to further optimize the extraction process. For instance, the study by Leite and colleagues [84] demonstrated that combining SUPRAS with microwave radiation significantly enhances the extraction rates of carotenoids and phenols. The key advantage of this combination lies in its ability to reduce extraction time while increasing efficiency, all while preserving the integrity of bioactive compounds [84].

While CPE has also been coupled with microwave-assisted extraction (MAE) to improve extraction yields, SUPRAS offers greater versatility and flexibility in solvent selection, making it particularly attractive for industrial-scale applications [77,78,79,80,81]. Supporting this, studies [81,82,83] have shown that CPE achieves high yields of phenols and flavonoids from peaches, bioactive compounds widely utilized in cosmetics and functional foods.

Recent research by Patel and colleagues [79] and Giovanoudis and colleagues [83] further confirms the superiority of CPE for extracting bioactive compounds from mandarin and pomegranate peels, yielding higher levels of phenolic compounds and flavonoids than traditional methods. Additionally, the study by Liew and colleagues [85] demonstrated that CPE extraction of antioxidant compounds from citrus fruits yields promising results in polyphenol and flavonoid recovery, highlighting its potential for applications in cosmetics and functional foods. Patel and colleagues [79] also emphasized that CPE-based extraction of mandarin bioactives resulted in exceptionally high flavonoid yields with significant antioxidant activity, reinforcing its suitability for future applications in the food industry and nutritional supplements.

Both CPE and SUPRAS represent eco-friendly and efficient alternatives to conventional extraction methods, particularly for reducing toxic solvent use and minimizing environmental impact [76,77]. Advances in experimental conditions—such as optimizing surfactant concentrations, pH modulation, and temperature adjustments—have enabled the efficient extraction of bioactive compounds from previously underutilized fruit residues, including peels and seeds [84]. However, some limitations persist, particularly regarding the scalability of these techniques for large-scale production, as well as challenges related to solvent recovery and reuse in SUPRAS [76,77]. Moving forward, integrating these techniques with other sustainable methodologies, such as supercritical CO_2_ extraction or laser-assisted extraction, could further enhance bioactive compound recovery while minimizing environmental impact [80]. The future perspectives for these techniques are promising, with expected advances in the recovery of bioactive compounds from fruits under more sustainable and cost-effective conditions [83]. The development of new surfactants and supramolecular solvents, as well as the improvement of experimental conditions, will likely contribute to advancing scalability for industrial applications. Additionally, the integration of hybrid techniques for bioactive compound extraction from fruits is expected to result in even more efficient and sustainable processes, with substantial potential for applications in the food, pharmaceutical, and cosmetic industries.

### 3.2. Comparative Analysis of Extraction Methods

The rapid evolution of extraction technologies necessitates a systematic comparison of their advantages, limitations, and applications [65]. A thorough understanding of these differences is essential for researchers and industry professionals to select the most suitable method for specific applications, considering factors such as cost-effectiveness, environmental impact, and scalability [50,66].

**Figure 2:**
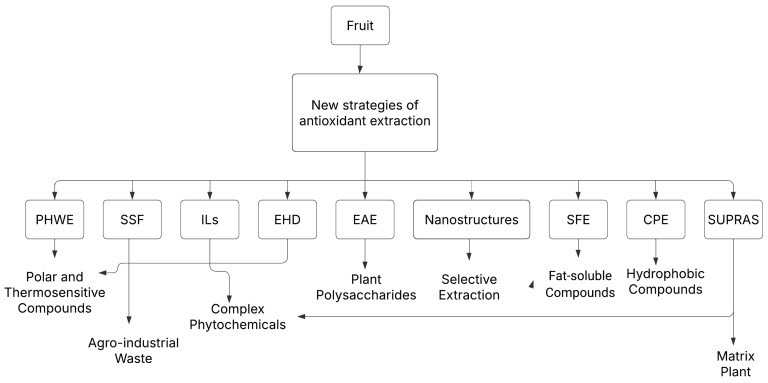
Extraction methods and their targeted bioactive compounds. Note: The figure shows the extraction methods and the compounds each technique targets, highlighting the suitability of PHWE and SSF for polar compounds, and SFE and SUPRAS for lipophilic and hydrophobic compounds.

Recent advancements in extraction methodologies have demonstrated varying degrees of efficiency and applicability across different fruit matrices. Figure 2 illustrates the extraction methods and the bioactive compounds targeted by each technique. Methods such as PHWE and SSF are effective for polar and thermosensitive compounds, while techniques like SFE and SUPRAS are more suited for lipophilic and hydrophobic compounds. Each technique offers distinct advantages and operational constraints that must be carefully assessed in relation to specific extraction objectives [50,66]. Table 1 provides a comparative overview of the primary extraction methods discussed in this review, outlining their key characteristics, operational parameters, advantages, limitations, and typical applications. This analysis highlights the specific benefits and limitations of each technique, allowing for a more informed selection based on the nature of the target compounds and the characteristics of the raw material [50].

In terms of adaptability, pressurized hot water extraction (PHWE) has proven particularly effective for extracting phytochemicals from various fruit parts. Its ability to process diverse plant materials highlights its versatility and efficiency. PHWE is especially advantageous for high-moisture materials, such as fresh fruits, where its solvent-free approach preserves compound stability while achieving high extraction yields [86]. Conversely, ionic liquid extraction demonstrates superior performance when applied to dried fruit powders, as its unique solvent properties facilitate better matrix penetration and enhanced compound recovery [87].

The processing of waste materials presents unique challenges that influence method selection. Solid-state fermentation (SSF) is particularly advantageous in this context, as it not only enables the extraction of bioactive compounds but also promotes biotransformation processes that enhance the antioxidant content of the final product [88]. SSF is widely employed in enzyme production, including pectinases, amylases, and lipases, with applications spanning the food, beverage, paper, and biofuel industries [19,23]. A key example is the production of cellulases for biomass conversion into bioethanol, where these enzymes facilitate cellulose degradation into fermentable sugars [24]. This dual functionality makes SSF particularly valuable for industrial applications focused on waste valorization [89].

Essential oil recovery also requires tailored methodological approaches based on the characteristics of the source material. Electrohydrodynamic extraction has demonstrated exceptional efficiency with citrus peel matrices, minimizing thermal degradation while preserving extraction yields [90]. Supercritical fluid extraction (SFE) is particularly effective for nonpolar compounds, such as essential oils and lipids, due to the unique properties of supercritical CO_2_ [91]. However, its high equipment costs and the frequent need for co-solvents for polar compound extraction remain significant limitations [91].

For seed-based materials, nanostructure-assisted extraction enhances selectivity and yield, particularly when isolating volatile compounds [92]. Additionally, SSF improves protein accessibility through controlled enzymatic hydrolysis [93], while nanostructure-assisted extraction enables the precise isolation of specific peptide fractions within complex matrices [94].

Cloud Point Extraction (CPE) and SUPRAS extraction techniques represent significant advances in strategies for extracting bioactive compounds from fruits, offering more ecological and efficient alternatives compared to traditional methods. However, the choice between these techniques depends on the specific needs of the application, including the complexity of the sample matrix, target compounds, and available resources [84]. While CPE is more accessible and straightforward, SUPRAS offers greater selectivity and efficiency, making it suitable for cases where precision in extracting specific compounds is crucial. Therefore, the adoption of these techniques should be carefully evaluated, considering their benefits, challenges, and limitations in relation to the final extraction objective [80].

Bitwell and colleagues [91] emphasize that modern extraction techniques generally offer higher efficiency, shorter processing times, and reduced solvent consumption, aligning with green chemistry principles. Their study underscores the importance of optimizing key extraction parameters, including solvent composition, temperature, pressure, and extraction duration, as these variables critically influence both efficiency and selectivity in bioactive compound recovery [91].

In some cases, hybrid approaches combining PHWE with subsequent ionic liquid extraction have demonstrated synergistic benefits, maximized recovery rates while preserving compound integrity [95]. Ionic liquid extraction is particularly advantageous for processing fruit pulp, as its mild extraction conditions help maintain bioactivity [27,28,29,30]. Furthermore, enzyme-assisted extraction (EAE) has been identified as a complementary technique that enhances yield by breaking down complex plant matrices, thereby improving the solubility and accessibility of target phytochemicals [91].

### 3.3. Industrial and Enviromental Implications, and Future Research Directions

While this study provides a comprehensive evaluation of extraction techniques, future research should prioritize the development of hybrid approaches that integrate multiple methodologies to maximize efficiency. Additionally, exploring novel solvent systems could offer more sustainable alternatives for phytochemical extraction.

Consequently, several extraction techniques have already been adopted in the industrial sector, with PHWE standing out as a highly efficient and environmentally sustainable method for obtaining green tea and caffeine extracts [96]. This technique effectively preserves the structural integrity of thermosensitive compounds, ensuring the production of high-quality extracts suitable for applications in functional foods, dietary supplements, and pharmaceuticals [96]. Specifically, PHWE has been successfully implemented for large-scale caffeine extraction, enabling the selective isolation of this alkaloid from sources such as coffee beans and tea leaves [96].

The hop extract utilized in the brewing industry is produced through supercritical fluid extraction (SFE) [97]. Among these, supercritical carbon dioxide (CO_2_-SC) is the most employed technique for isolating key aromatic and bitter compounds from hops, including alpha acids and essential oils, which play a fundamental role in beer flavor profile development [97]. This method is favored due to its ability to preserve sensory attributes while minimizing thermal degradation, thereby ensuring both the aromatic integrity and long-term stability of the final product [97].

Solid-state fermentation (SSF) has been extensively applied in the large-scale production of biotechnological, enzymatic, and bioactive compounds [23]. Its primary application lies in the synthesis of industrial enzymes, particularly hydrolytic enzymes such as cellulases, amylases, proteases, and lipases, which play a pivotal role in multiple industries [23]. These biocatalysts are crucial for various sectors, including food processing, biofuel production, detergent formulation, textile manufacturing, and pharmaceutical development [23].

SUPRAs and CPE represent highly efficient strategies for environmental monitoring, demonstrating broad applicability in the extraction of heavy metals and organic pollutants [98,99]. CPE is particularly advantageous for the selective extraction and preconcentration of heavy metals such as lead (Pb), cadmium (Cd), and mercury (Hg) from aqueous, soil, and sediment matrices [99]. In contrast, SUPRAS exhibits remarkable efficiency in the extraction of pesticide residues, particularly organochlorine and organophosphate compounds, which pose significant risks to water quality and food safety [98]. Both methodologies offer superior selectivity, sensitivity, and environmental sustainability, reinforcing their critical role in analytical chemistry for ecological risk assessment and public health protection [98,99].

Ionic liquid (IL) and nanostructure-assisted extraction methods have emerged as promising approaches in the large-scale production of cannabinoids, particularly cannabidiol (CBD) and tetrahydrocannabinol (THC) [100,101,102], which are widely recognized for their therapeutic and commercial potential. The integration of supercritical fluid extraction (SFE) with ILs has been investigated by Kornpointner and colleagues [101], who reported the first application of IL-based dynamic supercritical CO_2_ extraction for the selective recovery of six cannabinoids—CBD, CBDA, Δ9-THC, THCA, CBG, and CBGA—from industrial hemp (*Cannabis sativa* L.). This innovative methodology exhibits a synergistic effect, enhancing extraction efficiency while eliminating the need for conventional organic solvents [100,101,102]. These advanced extraction strategies are now well-established and offer significant scalability, reinforcing their applicability in the pharmaceutical and nutraceutical industries [100,101,102].

Scale considerations play a crucial role in the selection of methods for industrial applications [50]. At the laboratory scale, all methods demonstrate viability, with selection primarily influenced by compound characteristics and research objectives [9,50]. However, as operations scale up to pilot and industrial levels, pressurized hot water extraction (PHWE) and solid-state fermentation (SSF) demonstrate superior scalability and cost-effectiveness [103]. These techniques remain commercially viable, although emerging technologies continue to advance towards industrial applicability [11]. Economic analyses show significant variations in operational costs across different methods [50]. While ionic liquid and nanostructure-assisted extractions tend to incur higher operational expenses, their use may be justified for high-value compounds where purity and selectivity requirements outweigh cost considerations [27,28,29,30]. In contrast, PHWE and SSF consistently offer more favorable economic outcomes for bulk extraction processes, particularly in the valorization of agricultural waste materials [104].

The adoption of green extraction technologies aligns with sustainability objectives by reducing solvent waste and energy consumption [91]. From an industrial perspective, these findings have significant implications for the food, pharmaceutical, and cosmetic sectors. Furthermore, the scalability of these modern techniques must be further investigated to ensure their feasibility for large-scale production.

The environmental impact of solvent use remains a critical consideration [91]. Techniques such as PHWE and SFE offer solvent-free or low-solvent alternatives, thereby reducing hazardous waste generation [91]. However, challenges related to solvent recovery and the economic feasibility of these methods must be addressed to facilitate their widespread adoption [91].

The scalability of supercritical fluid extraction (SFE) presents several technical and economic challenges [61]. The need for high-pressure equipment and precise operational conditions significantly increases production costs [61,62]. Additionally, the energy demands of maintaining supercritical conditions may limit the feasibility of large-scale operations, particularly in regions with high energy costs [62]. Although the reuse of CO_2_ is advantageous, the continuous compression and recycling processes require sophisticated infrastructure and monitoring systems [61,62,63]. Furthermore, regulatory compliance and the need for specialized personnel for operation and maintenance add further barriers to industrial implementation [62]. Developing cost-effective and energy-efficient extraction systems, along with optimizing process integration into existing production lines, will be critical for expanding the commercial use of SFE [62].

Scaling up nanostructure-assisted extraction from laboratory settings to industrial applications presents several challenges. The synthesis and functionalization of nanostructures at large scales require high precision and consistency, which can be difficult to achieve cost-effectively [58]. Additionally, the recovery and reuse of nanomaterials remain major concerns, as their separation from extraction media can be technically demanding [58]. The integration of these advanced materials into existing industrial processes also requires significant adaptation, increasing initial investment costs [56,57]. Moreover, ensuring batch-to-batch reproducibility in large-scale production is critical for maintaining the efficiency and reliability of the extraction process [55,56,57]. Overcoming these scalability challenges will require further technological advancements, optimization of synthesis protocols, and the development of cost-effective, sustainable solutions [58].

One of the primary obstacles to enzyme-assisted extraction (EAE) is the cost and availability of enzymes, as large-scale applications require significant quantities of highly purified enzymes, which may not always be economically viable [45]. Additionally, the reproducibility of enzymatic reactions in large batches can be difficult due to variations in raw material composition and enzyme stability [47]. The need for controlled reaction conditions, such as pH and temperature, further complicates the scalability of this technique [45,46,47]. Furthermore, enzyme recovery and reuse remain major concerns, as most enzymes are inactivated after a single use, increasing production costs and generating waste [45,46,47]. Addressing these challenges will require advancements in enzyme immobilization, process optimization, and the integration of EAE with other green extraction technologies.

The integration of computational tools, such as artificial neural networks (ANNs) and multi-objective optimization (MOO), has emerged as a powerful strategy for optimizing extraction conditions [91,105]. By modeling experimental data, these approaches enable the precise prediction of optimal extraction parameters, significantly reducing the reliance on trial-and-error experimentation [105].

ANN models are capable of processing large datasets and identifying complex non-linear relationships between variables, resulting in highly accurate predictions [91]. Meanwhile, MOO provides a structured framework for balancing multiple extraction objectives, such as maximizing yield while minimizing solvent consumption and energy input [91]. The combined application of these computational techniques enhances process efficiency and ensures reproducibility in large-scale operations [91,105]. Future advancements in machine learning algorithms and real-time monitoring systems are expected to further refine extraction optimization, reinforcing the indispensable role of these approaches in modern phytochemical research [91,105].

Additionally, the stability and bioavailability of extracted compounds should be further investigated to ensure their efficacy in pharmaceutical and nutraceutical applications. The implementation of real-time monitoring techniques, such as spectroscopy and chromatography, could enhance process control and improve reproducibility in industrial settings [105].

## 4. Conclusions

The transition to modern, sustainable extraction technologies is essential for advancing natural product research while minimizing environmental impact. Future developments in extraction science should integrate optimization strategies and computational modeling to enhance efficiency and promote eco-friendly methodologies for bioactive compound recovery. This article provides a comprehensive overview of recent advancements in natural product extraction techniques, emphasizing the shift from conventional solvent-based methods to more sustainable and efficient approaches. In addition to summarizing recent innovations, it highlights the comparative effectiveness and industrial scalability of emerging techniques, underscoring the need for systematic evaluations that consider different fruit matrices, target compounds, and economic feasibility. Addressing these gaps will be crucial for translating laboratory-scale innovations into commercially viable extraction processes.

Among the emerging techniques, pressurized hot water extraction (PHWE) and electrohydrodynamic (EHD) extraction have demonstrated significant potential for recovering antioxidants and thermosensitive compounds from various natural sources. Solid-state fermentation (SSF) has gained recognition as an effective strategy for valorizing agro-industrial waste and repurposing discarded biomaterials. Ionic liquids (ILs) offer a promising alternative for the selective extraction of complex phytochemicals; however, challenges related to scalability and economic feasibility must be addressed to enable their broader industrial adoption. Additionally, Cloud Point Extraction (CPE) has gained attention as an environmentally friendly approach that utilizes the phase separation of nonionic surfactants to selectively extract bioactive compounds, reducing solvent consumption and improving process sustainability. Similarly, supramolecular solvent extraction (SUPRAS) has emerged as a promising method for efficiently isolating phytochemicals by leveraging self-assembled nanostructures, offering high selectivity and versatility in extracting a wide range of bioactive molecules.

These innovative methodologies represent a paradigm shift toward more efficient and sustainable extraction processes, enabling the discovery of high-value bioactive compounds and driving advancements across multiple industries. Future research should focus on overcoming current limitations through technological innovations, including process automation, real-time monitoring, and the development of cost-effective, environmentally friendly solvents. The successful integration of these techniques into industrial, food, and pharmaceutical applications will not only enhance extraction efficiency but also contribute to a more sustainable approach to utilizing natural resources for phytochemical and antioxidant production.

## Figures and Tables

**Table 1:** Comparison of Novel Extraction Methods for Fruit-Based Bioactive Compounds.

Method	Key Advantages	Limitations	Typical Applications	Operating Conditions
Pressurized Hot Water Extraction (PHWE)	- Environmentally friendly - No organic solvents - High extraction efficiency	- High energy consumption - Possible degradation of heat-sensitive compounds - High equipment costs	- Phenolic compounds - Antioxidants - Water-soluble compounds	- Temperature: 60–120 °C - Pressure: 10–80 bar
Solid-State Fermentation (SSF)	- Low cost - Valorization of waste - Natural process	- Long processing time - Complex parameter control - Scale-up challenges	- Enzyme production - Bioactive compounds - Secondary metabolites	- Temperature: 25–35 °C - Humidity: 40–70% - Time: 3–15 days
Ionic Liquid Extraction	- High selectivity - Tunable properties - Good stability	- High cost of ionic liquids - Recovery complexity - Potential toxicity concerns	- Specific target compounds - Heat-sensitive materials - Complex matrices	- Room temperature to 80 °C - Atmospheric pressure
Electrohydrodynamic Extraction	- Rapid extraction - Low temperature operation - High efficiency	- Equipment complexity - Limited scale-up data - High initial investment	- Thermolabile compounds - Essential oils - Antioxidants	- Voltage: 14–22 kV - Time: 2–30 min
Nanostructure-Assisted Extraction	- Enhanced selectivity - Improved efficiency - Lower solvent usage	- Cost of nanoparticles - Recovery challenges - Limited commercial scale	- Targeted compounds - Trace elements - Specific metabolites	- Varies by nanostructure type
Supercritical Fluid	- Specific extraction of compounds - Uses recyclable CO_2_ - Prevents thermal degradation - Produces extracts with minimal impurities	- Expensive equipment - Low solubility of polar compounds - High energy consumption	- Extracting oils, flavors, and pigments - Bioactives and antioxidants - Modifying polymers and extracting additives	- Temperature: 30–80 °C - Pressure: 80–500 bar
Surfactant-Based Extraction: CPE and SUPRAS	- High affinity for bioactive compounds - Selectivity and Efficiency - Low Energy and Cost-Effective - Versatility in Bioactive Compound Extraction	- Surfactant Residues - Limited Solubility Range -Scalability - Phase Separation Challenges - Potential alterations in bioactivity due to surfactant interactions	- Recovery of antioxidants and essential oils - Extraction of polyphenols, carotenoids, and flavonoids - Removal of heavy metals, pesticides, and organic pollutants	-Temperature: 25–80 °C - pH: 3–8

Note: The key advantages and limitations of the method were evaluated in comparison with traditional extraction methods, highlighting the improvements and trade-offs associated with each approach.

## Abbreviations

The following abbreviations are used in this manuscript:

TPC	Total Phenolic Content
DPPH	2,2-difenil-1-picril-hidrazila (2,2-Diphenyl-1-picrylhydrazyl)
ABTS	Ácido 2,2′-azino-bis(3-etilbenzotiazolina-6-sulfônico) (2,2′-azino-bis(3-ethylbenzothiazoline-6-sulfonic acid))
FRAP	Ferric Reducing Antioxidant Power
PHWE	Pressurized Hot Water Extraction
IC50	Half Maximal Inhibitory Concentration (50%)
CEAC	Vitamin C Equivalent Antioxidant Capacity
SSF	Solid-state fermentation
TAA	Total Antioxidant Activity
TFC	Total Flavonoid Content
BP	Bound Polyphenols
GAE	Gallic Acid Equivalents
IL	Ionic Liquids
ABS	Aqueous Biphasic System
PPG400	Polypropylene Glycol 400
ATPS	Aqueous Two-Phase System
EHD	Electrohydrodynamic-assisted extraction
AA	Antioxidant Activity
EE	Extraction Efficiency
EAE	Enzyme-assisted extraction
CUPRAC	Cupric-Reducing Antioxidant Capacity
SFE	Supercritical fluid extraction
HD	Hydro distillation
PLE	Pressurized Liquid Extraction
SCWE	Subcritical Water Extraction
SE	Soxhlet extraction
CPE	Cloud Point Extraction
SUPRAS	Supramolecular Solvent Extracion
MOFs	Metal-organic Frameworks
MOO	Multi-objective Optimization
ANN	Artificial Neural Networks
CBD	Cannabidiol
THC	Tetrahydrocannabinol
